# Public representations of the nursing image in YouTube comments: analysis based on Jean Watson’s theory

**DOI:** 10.1590/1980-220X-REEUSP-2025-0346en

**Published:** 2026-04-27

**Authors:** Edgardo Álvarez-Muñoz, Fernando Malhue-Torres, Catalina Sepúlveda-Rivas, Rocío Zúñiga-Tapia

**Affiliations:** 1 Universidad San Sebastián, Facultad de Ciencias para el Cuidado de la Salud, Escuela de Enfermería, Concepción, Chile.; 2 Universidad Santo Tomás, Facultad de Salud, Escuela de Enfermería, Temuco, Chile.; 3 Universidad Andrés Bello, Facultad de Enfermería, Escuela de Enfermería, Concepción, Chile.

**Keywords:** Social Network Analysis, Nursing, Communications Media, Social Networking, Análise de Rede Social, Enfermagem, Meios de Comunicação, Rede Social

## Abstract

**Objective:**

To analyze how the nursing image, present in public comments on YouTube videos that broadcast news, reports, or articles related to the profession in four television channels in Chile, is publicly represented and symbolically constructed.

**Method:**

A qualitative study based on content analysis, according to the guidelines of Elo and Kyngäs. A total of 1,018 public comments from 13 YouTube videos were analyzed. Purposive sampling and inductive thematic analysis were applied using ATLAS.ti ^®^ software. The interpretation of the findings was furthered using Jean Watson's Theory of Human Caring. Ethical approval was not required, but the commentators’ identity was protected.

**Results:**

Thematic analysis identified two categories: public representations that violate the ethics of humane care in nursing and public representations that reinforce it.

**Conclusion:**

The findings reveal a polarization between discourses of stigmatization and those of ethical and human recognition. These tensions reflect challenges in the social positioning of nursing and in the appreciation of care as the core of practice.

## RESUMO

**Objetivo:**

Analisar como a imagem da enfermagem, presente em comentários públicos em vídeos do YouTube que transmitem notícias, reportagens ou artigos relacionados à profissão em quatro canais de televisão no Chile, é representada publicamente e construída simbolicamente.

**Método:**

Estudo qualitativo baseado em análise de conteúdo, segundo as diretrizes de Elo e Kyngäs. Um total de 1.018 comentários públicos de 13 vídeos do YouTube foram analisados. Foram aplicadas amostragem intencional e análise temática indutiva utilizando o software ATLAS.ti ^®^. A interpretação dos resultados foi aprofundada utilizando a Teoria do Cuidado Humano de Jean Watson. Não foi necessária aprovação ética, mas a identidade dos comentaristas foi protegida.

**Resultados:**

A análise temática identificou duas categorias: representações públicas que violam a ética do cuidado humanizado em enfermagem e representações públicas que a reforçam.

**Conclusão:**

Os resultados revelam uma polarização entre os discursos de estigmatização e os de reconhecimento ético e humano. Essas tensões refletem desafios no posicionamento social da enfermagem e na valorização do cuidado como o cerne da prática.

## INTRODUCTION

Nurses represent the largest professional segment of the workforce within healthcare systems^(1,2)^, being recognized for both their caregiving role and their leadership capacity in complex healthcare contexts^(3)^. Their work gained greater media exposure following the COVID-19 pandemic, during which nursing staff gained greater visibility in the media^(3,4,5)^, and they were highlighted as key figures in the healthcare response, which reinforced their presence on the public agenda and in discourses about care.

The expansion of digital media and social networks, particularly platforms like YouTube, has opened a new space for the expression of public opinions on issues of social interest. This transformation has driven interest in conducting research on health topics through the analysis of comments on social media and virtual environments^(6,7,8)^. In this context, digital platforms function not only as communication channels but also as spaces for symbolic production where social narratives are shaped around different actors and professions.

Even though there are studies that analyze the image of nursing in the media^(9,10)^, there is scarce research addressing this phenomenon from the perspective of user-generated content on digital platforms. This gap restricts understanding of how public representations of nursing are configured in the digital space, as well as of the discourses that circulate around the profession.

Belting defines the concept of image as a personal or collective symbolization that has been constructed in the human minds based on the social imaginary of the moment projected by a person or a group^(11)^. This professional image, in the case of nurses, has been evolving in a dynamic influenced by several factors: from daily contact with patients and other professionals to the creation of a body of own knowledge based on the advance of practice based in evidence^(12)^.

This evolution does not follow a linear trajectory nor is it homogeneous, but rather responds to historical, cultural, and political contexts that influence the way the profession is perceived by society.

In the field of social media communication, user interaction with this content (engagement) can reflect levels of emotional, critical, or evaluative involvement, which help identify symbolic and affective elements associated with the professional image of the nurse^(13)^. Therefore, in line with approaches examining public representations and shared meanings constructed in everyday digital communication, the comments users leave in videos related to nursing allow to explore perceptions, prejudices, assessments, and representations that not always emerge in surveys or other traditional qualitative methods. This type of spontaneous content facilitates access to the everyday dimension of social thought, where shared meanings are configured, often implicit, affecting the way the work of nurses is understood and valued.

In this sense, this study contributes to identifying stereotypes, assessing the social impact of media narratives, and making the professional image of nurses in the contemporary digital environment visible.

This type of analysis is crucial for understanding how the social image of the profession is configured in the digital space and what implications it could have in terms of new relationships with society. Furthermore, it allows us to identify tensions between the institutionally promoted image and the public perceptions freely expressed on social media, which opens opportunities to rethink communication and educational strategies from the nursing field.

Consequently, and considering the concept of image proposed by Hans Belting^(11)^ (a socially constructed symbolization), this study seeks to analyze the public representations of nursing’s image expressed in comments on YouTube videos that broadcast news, reports, or articles related to the profession, published by four broadcast television channels in Chile.

This analysis is complemented by Jean Watson’s^(14)^ theory of human care, which proposes that care is not only a technical act, but a moral and human commitment to the other, which includes respect, sensitivity and authentic presence. From this perspective, caring also implies safeguarding the dignity of the profession, given that the relationship between nursing and society is part of the care environment. Thus, understanding how the image of the profession is shaped in digital spaces allows us to critically reflect on the ethical, cultural and communicational conditions that favor or distort the recognition of care as an essential value of nursing practice^(14)^.

## METHOD

### Type or Design of Study

Qualitative study based on inductive content analysis, with interpretative support from Jean Watson’s Theory of Human Caring^(14)^.

### Data Collection

Data collection was carried out through public comments made in reports, news and videos related to nursing, published on the YouTube accounts of four Chilean television channels: Televisión Nacional de Chile (TVN), Canal 13 (C13), Chilevisión (CHV) and Mega, which were intentionally selected since they are the main open television channels, with national reach and those with the largest audience in the country^(15)^.

YouTube was chosen as the analysis platform because all channels have active accounts on this network, it allows for longer video uploads compared to other social networks such as Instagram, and allows for the reading of comment threads that remain visible and accessible to the public. Furthermore, the demographic profile of users of content on this social plat­form is varied, as it is used and preferred by people of different generations^(13)^.

### Selection Criteria and Sample Definition

The videos were selected through a YouTube search using the term “nursing” in Spanish, combined with the abbreviated name of each of the four television channels, along with the word “Chile” in the case of two stations, to avoid confusion with channels of the same name from other countries. Variants of the term “nursing” were accepted in the video titles, considering gender and grammatical number, excluding those that focused on other professions, such as nursing technicians or assistants.

The first 20 results that the platform displayed by default were considered for the selection, based on their thematic relevance and the highest likelihood of being viewed by users.

Of these videos, the four with the highest number of comments were identified, under the assumption that they generated the greatest audience interest. Only one of the channels (C13) had just one video included, as the others did not feature comments. No exclusion criteria related to video content were applied, with the aim of promoting thematic diversity of the analyzed comments.

Comments were extracted using the web-based software *Export Comments* ^®^ (https://exportcomments.com/), exporting them to *Microsoft Excel* ^®^ format. They were subsequently anonymized and stored on the *Mendeley Data* ^®^ platform, where they are available for viewing in their original language^(16)^.

### Analysis

For data analysis, the study used an inductive thematic analysis approach, based on the methodology proposed by Elo and Kyngäs^(17)^. Following the stages defined by the authors, the preparation phase began with the selection of the units of analysis, corresponding to the comments published in news and YouTube reports; during this phase, two researchers repeatedly read the comments, assigning meaning, relevance, and value to each unit of analysis.

In the organization phase, an inductive content analysis approach was used, which included open coding, category creation, and data abstraction. In open coding, both researchers jointly read the comments, taking notes and generating preliminary categories based on emerging themes. Subsequently, repetitive categories were eliminated, and the remaining ones were grouped into similar or higher-level categories, giving rise to subcategories. These subcategories allowed the phenomenon to be described, have its understanding broadened, and contributed to knowledge generation. For abstraction, the researchers assigned a name and a general description to the main consolidated categories. According to Elo and Kyngäs^(17)^, this phase should be extended as far as is reasonable and feasible; therefore, the researchers reached a consensus to determine the endpoint of the abstraction process.

The entire process of coding, organizing, and analyzing the data was conducted using ATLAS.ti^®^ version 25, which enabled the systematization of textual information, the organization of emerging categories, and the facilitation of qualitative traceability. It is important to note that, at the end of the analysis process, the researchers improved the writing and grammar and adapted local idioms present in the comments without altering their meaning, to facilitate comprehension.

Although the thematic analysis was carried out using an inductive approach, the interpretation of the findings was further enriched with elements of Jean Watson’s Theory of Human Caring^(14)^. This framework allowed for a deeper understanding of the ethical, relational, and human dimensions of the identified representations, enabling a critical reading of how the social discourses expressed in the comments reinforce or erode fundamental values of care—such as empathy, dignity, sensitivity, and authentic presence—which are considered central to nursing practice.

This research met the Consolidated Criteria for Reporting Qualitative Research (COREQ)^(18)^.

### Ethical Aspects

Due to the research methodology employed and the public nature of both YouTube and the comments analyzed, the study did not require approval from a Scientific Ethics Committee. However, to protect the identity of the commenters, the researchers anonymized the profiles and did not contact them.

## RESULTS

This section presents the findings of the thematic analysis of comments made on nursing-related videos from Chile’s four main broadcast television channels. A total of 13 videos were included, from which 1018 comments were extracted and subjected to analysis. The characteristics of the included videos are described in Table 1.

**Table 1:** YouTube videos included by open television channel – Chile, 2025.

Search strategy	Video title in original language	Links	Comments	Export date
“Enfermería TVN Chile”	Enfermero formalizado por brutal golpiza a conserje	https://www.youtube.com/watch?v=80Mz3nIS7h0	n = 30	06/12/2025
	Federación de enfermeros pide cambiar el manejo de la pandemia: "estamos sobreexplotados"	https://www.youtube.com/watch?v=4tCq_Bi6d3A	n = 33	06/12/2025
	Denuncian que enfermera utilizó la misma jeringa para vacunar a varias personas	https://www.youtube.com/watch?v=rpyXHT3yfEs&t=2s	n = 33	06/12/2025
	Grave agresión: Paciente habría tosido en la cara de enfermera	https://www.youtube.com/watch?v=MdeQY1hXDbQ	n = 56	06/12/2025
“Enfermería CHV”	FISCAL ENTREGA DETALLES tras detención de sospechosos de ataque a enfermera	https://www.youtube.com/watch?v=BxihWaDwOcY	n = 93	06/12/2025
	Habla Pola Álvarez, la enfermera apuñalada en Las Condes: “Recuerdo todo”	https://www.youtube.com/watch?v=OQuH1in1NJA	n = 116	06/12/2025
	REVELAN CONVERSACIÓN clave entre imputados previo al ataque a enfermera: “La Pola es esa, la rucia”	https://www.youtube.com/watch?v=3QHygTGQsu0	n = 142	06/12/2025
	IMPACTANTE VUELCO: Ex funcionaria y su pareja detenidos por ataque a enfermera	https://www.youtube.com/watch?v=837A5WXJ_Y0	n = 246	06/12/2025
“Enfermería Mega”	¡Heroicas! Enfermeras salvan a recién nacidos en terremoto	https://www.youtube.com/shorts/Szd2mAMPZmw	n = 8	06/12/2025
	Personalidad narcisista: El perfil de la enfermera imputada por crimen de Pola Álvarez	https://www.youtube.com/watch?v=2slYQsqNIH8	n = 9	06/12/2025
	Dos atacantes de enfermera son declarados culpables	https://www.youtube.com/watch?v=PMi1gb2t81w	n = 16	06/12/2025
	Conmovedor mensaje de enfermera emociona a ministros y Presidente en La Moneda	https://www.youtube.com/watch?v=8OrbfU001YY	n = 205	06/12/2025
“Enfermería Canal 13 Chile”	Enfermera chilena en Italia y la lucha contra el coronavirus	https://www.youtube.com/watch?v=vTo5E_NUf88	n = 31	06/12/2025

**Figure 1:**
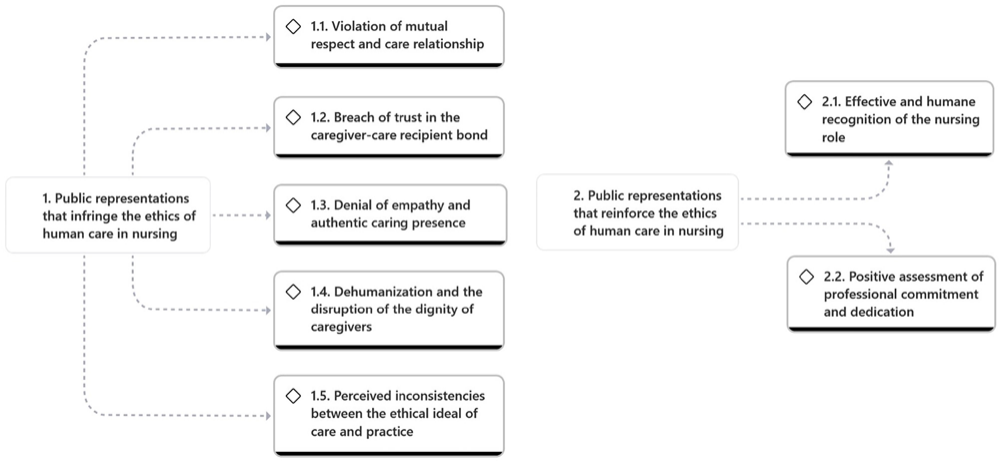
Conceptualization of the categories and subcategories emerging from content analysis.

Two main categories were identified from the thematic analysis: Public representations that violate the ethics of humane care in nursing and public representations that reinforce the ethics of humane care in nursing. The categories presented below reflect patterns of meaning emerging from public discourse expressed in YouTube comments. These categories do not represent theoretical social representations, but rather public representations and evaluative constructions identified through inductive content analysis. The emerging categories and subcategories are represented in Figure 1.

### Public Representations that Infringe the Ethics of Human Care in Nursing

From the perspective of transpersonal care and the ethics of care proposed by Jean Watson, these representations show a disconnect between the fundamental principles of professional care and the social images circulating in digital spaces. Watson conceives care as a moral and relational act expressed through empathy, compassion, respect for dignity, and authentic presence. Therefore, public representations that dehumanize nursing professionals signal a rupture in the relationship between care and culture. Five subcategories emerged from this category.

#### Violation of Mutual Respect and Care Relationship

Watson argues that authentic care is grounded in respect and equality^(14)^. In this case, the comments reinforce an authoritarian image that contradicts the ethical ideal of care.

*“Remove her title for lack of ethics and aggressiveness” (TVN)*

*“Nursing is a profession of egos and abuse toward new people” (CHV)*

*“They criticize and discredit each other, completely ruining our professionalism” (CHV)*

#### Breach of Trust in The Caregiver-Care Recipient Bond

Trust is a key component in Watson’s^(14)^ transpersonal care process. This subcategory shows how social discourse instills fear, undermining that fundamental bond.

*“Revoking his degree is a danger to society” (TVN)*

*“He doesn’t know how to control his anger; that man can’t care for patients” (TVN)*

*“This incident is highly dangerous because it involves people who are “prepared or qualified” to work in the healthcare field” (CHV)*

#### Denial of Empathy and Authentic Caring Presence

Watson defends authentic presence as the basis of humane caring^(14)^. This subcategory groups together representations that deny this essential quality, associating nursing with coldness, indifference, or dehumanization.

*“They are cold-blooded because they are indifferent to the sick” (CHV)*

*“To think that these “people” provide care in hospitals and clinics” (MEGA)*

*“Nurses are sometimes indolent and dehumanizing” (CHV)*

#### Dehumanization and The Disruption of the Dignity of Caregivers

Here, the professional is stripped of their ethical and human identity, which directly contradicts the foundations of Watson’s theory, which considers dignity an inalienable value of caregiving^(14)^.

*“A crazy woman, a patient manager, my God” (MEGA)*

*“How can any crazy criminal work with patients?” (CHV)*

*“That’s a criminal and a sick person... Not a nurse... He should be in jail” (TVN)*

#### Perceived Inconsistencies between the Ethical Ideal of Care and Practice

Here, the comments judge professional behavior based on an unmet ethical expectation. From Watson’s perspective, this can be interpreted as a disconnect between the expected care (based on ethics and humanity) and what is observed or socially represented^(14)^.

*“No professional ethics” (CHV)*

*“There is no professional ethics at all” (TVN)*

*“Professionals with ethics save human lives at all levels, good treatment, judgment, humanity, empathy... which in this case is totally contradictory” (CHV)*

### Public Representations that Reinforce the Ethics of Human Care in Nursing

From Watson’s perspective, these comments express a public appreciation of care as an ethical, relational, and profoundly human act. The discourses recognize nursing not only as a technical profession but as a moral commitment to others. Two subcategories emerged.

#### Effective and Humane Recognition of the Nursing Role

These meanings align with Watson’s view of caring as a moral, spiritual, and relational expression.

Care as an altruistic and ethical act, based on compassion and love for others. For Watson, caring is not only a professional action, but a spiritual and moral expression that honors the life of others^(14)^. Recognizing nurses as figures of dedication, protection, and courage is consistent with professionals who authentically connect with others, without expecting reward, promoting healing beyond the physical body.

*“They give their lives for people” (Mega)*

*“They work risking their lives to protect us” (TVN)*

*“A giant, brave heart, determined to give everything for others without expecting anything in return” (Mega)*

#### Positive Assessment of Professional Commitment and Dedication

Here, the public anchors nursing in values such as sensitivity, presence, and responsibility. These are shared cultural meanings that shape the collective imagination about the profession and resonate with the Caritas processes described by Watson. Ethical, technical, and emotional commitment implied by the professional practice of nursing. The reference to “being in the hands” of a nurse refers to the concept of authentic presence, in which the professional is completely available to care for others^(14)^. Likewise, “sensitivity” alludes to the central aspects of the Caritas process.

*“How can people imagine that a nurse is going to reuse syringes?” (TVN)*

*“Nursing staff who should have great sensitivity toward their fellow human beings” (CHV)*

*“I always admired nurses because my life was in their hands every time they inserted an IV” (CHV)*

## DISCUSSION

While there are studies that analyze the social image of nurses in traditional media^(19,20,21,22)^, new forms of digital interaction, such as websites, blogs and social networking platforms like YouTube and X, have acquired a relevant influence^(23)^. In this context, considering that 66% of the world’s population, that is, more than 5.350 million people are internet users^(24)^, this study offers a novel approach, focusing specifically on the analysis of user comments on a digital social network, which allows access to spontaneous and unmediated perceptions of the public.

Regarding the results, the negative representations, which were predominant, offer a stigmatized view of the professional image of nurses. This finding is consistent with a scoping review, which indicates that the media tend to predominantly disseminate a negative image of the profession^(10)^. This perception of the professional image constitutes a powerful mechanism that influences key aspects such as the self-esteem and authority of nurses, as well as the recruitment and retention of personnel in the health sector^(25,26,27)^ and even their intention to leave the profession^(28)^. Even the International Council of Nurses (ICN) highlighted the importance of this issue in its global report, which reflects how the lack of recognition and a deteriorated social image contribute to workforce shortages and high turnover worldwide^(29)^.

Conversely, positive representations of the professional image, although present, emerge less frequently and particularly in videos where nurses are represented as figures of heroism and sacrifice. Regarding this positive image, some authors maintain that, in general, society attributes greater importance to characteristics such as kindness and closeness, identifying vocation as the central core of nursing^(30)^. In line with this, Jean Watson suggests that professional care is based on an authentic disposition toward others, sustained by love, commitment and the intention to heal: “the act of caring is a moral and spiritual expression, not merely a technical one”^(14)^.

A positive image of a profession in society can confer power, recognition, and status, which contributes to the symbolic and moral positioning of the profession in society^(19)^. In terms of humane caring, such a public image also strengthens the ethical legitimacy of nursing, since, as Watson points out, “authentic caring is based on mutual respect and recognition of shared humanity”^(14)^. Therefore, presenting a positive public image of nursing is an urgent issue that must be addressed to create an empowered nursing workforce^(31)^ and with greater leadership capacity^(32)^.

Social trust in the nursing role, when eroded through stigmatizing representations, not only weakens the professional image, but, from a humanistic perspective, damages the essential bond between the caregiver and the recipient^(33)^. Watson argues that “trust is the foundation upon which every caring relationship is built” and that without it, the healing process is threatened, which contributes to the symbolic and moral positioning of the profession in society^(14)^. Although only one of many nursing theories reflecting caring, Watson’s emphasis on authentic relationships in healing environments reinforces the idea that nursing is not just a profession but a calling to serve humanity with empathy and love^(34)^.

In this challenging scenario, it is important that nurses and the professional organizations representing them generate instances of active interaction, with the purpose of projecting and transmitting a more realistic image of the profession^(35)^. By actively participating in media such as television channels, nurses can make their role, value and important contribution to health systems known, and reduce the transmission of content that negatively affects the image that society forms of the profession. However, for Watson, this task is not only communicative, but also ethical, since it implies making care visible as a human, relational and dignified practice, which “recognizes the other as a unique and unrepeatable being, deserving of respect, compassion and authentic presence”^(14)^.

Finally, despite its methodological rigor, this study has some limitations. These are mainly related to the characteristics of the selected videos, which mostly correspond to reports and news with a negative focus on the profession, and could have influenced the content of the analyzed comments. Furthermore, although the findings provide an initial insight into the public image of nursing on this platform, future research should be expanded to other social networks, such as Instagram, X, or Facebook, to further enrich the understanding of nursing’s social representation in the digital environment.

## CONCLUSION

This study allowed us to analyze the public representations and discourses circulating about nursing on a high-reach digital platform such as YouTube, based on spontaneous user comments on news related to the profession. The results reveal a coexistence of contradictory representations: on the one hand, a positive image linked to dedication, sensitivity, and ethical commitment, values deeply linked to humane care and widely considered by society; on the other, a predominant narrative of mistrust, stigmatization, and questioning of the professional role, which affects public trust and the nurses’ social status. This duality reflects persistent tensions in the public perception of the profession and opens a field for reflection on the social, cultural, and ethical factors that influence its recognition. In this context, it is essential to promote strategies that strengthen the visibility of care as an ethical and humane practice, restoring dignity and trust in nursing, as proposed by Jean Watson’s Theory of Human Caring, thus contributing to the empowerment and social appreciation of the profession.

## Data Availability

The entire dataset supporting the results of this study is available upon request to the corresponding author.
