# Supplementary material for: Public representations of the nursing image in YouTube comments: analysis based on Jean Watson’s theory
Source: Rev Esc Enferm USP. 2026 Apr 27;60:e20250346. doi: 10.1590/1980-220X-REEUSP-2025-0346en (PMC13120761; doi:10.1590/1980-220X-REEUSP-2025-0346en)
Supplement: Translation

# Translation: Representaciones públicas de la imagen de enfermería en comentarios de YouTube: análisis basado en la teoría de Jean Watson

**DOI:** 10.1590/1980-220X-REEUSP-2025-0346es

**Authors:** Edgardo Álvarez-Muñoz, Fernando Malhue-Torres, Catalina Sepúlveda-Rivas, Rocío Zúñiga-Tapia

**Affiliations:** 1 Universidad San Sebastián, Facultad de Ciencias para el Cuidado de la Salud, Escuela de Enfermería, Concepción, Chile.; 2 Universidad Santo Tomás, Facultad de Salud, Escuela de Enfermería, Temuco, Chile.; 3 Universidad Andrés Bello, Facultad de Enfermería, Escuela de Enfermería, Concepción, Chile.

**Keywords:** Análisis de Redes Sociales, Enfermería, Medios de Comunicación, Red Social

## Abstract

**Objetivo:**

Analizar cómo se representa públicamente y se construye simbólicamente la imagen de enfermería presente en los comentarios públicos de videos de YouTube que difunden noticias, reportajes o artículos relacionados con la profesión en cuatro canales de televisión de Chile.

**Método:**

Estudio cualitativo basado en el análisis de contenido, según las directrices de Elo y Kyngäs. Se analizaron 1.018 comentarios públicos de 13 vídeos de YouTube. Se aplicó muestreo intencional y análisis temático inductivo utilizando el software ATLAS.ti ^®^. La interpretación de los hallazgos se profundizó utilizando la Teoría del Cuidado Humano de Jean Watson. No se requirió aprobación ética, pero se protegió la identidad de los comentaristas.

**Resultados:**

El análisis temático identificó dos categorías: representaciones públicas que violan la ética del cuidado humano en enfermería y representaciones públicas que la refuerzan.

**Conclusión:**

Los hallazgos revelan una polarización entre los discursos de estigmatización y los de reconocimiento ético y humano. Estas tensiones reflejan desafíos en el posicionamiento social de la enfermería y en la valorización del cuidado como núcleo de la práctica.

## INTRODUCCIÓN

Las enfermeras representan el segmento profesional más numeroso de la fuerza laboral dentro de los sistemas de salud^(1,2)^, y son reconocidas tanto por su función asistencial como por su capacidad de liderazgo en contextos sanitarios complejos^(3)^. Su trabajo ganó mayor exposición mediática tras la pandemia de COVID-19, durante la cual el personal de enfermería adquirió mayor visibilidad en los medios de comunicación^(3,4,5)^ y fue destacado como figura clave en la respuesta sanitaria, lo que reforzó su presencia en la agenda pública y en los discursos sobre la atención sanitaria.

La expansión de los medios digitales y las redes sociales, en particular de plataformas como YouTube, ha abierto un nuevo espacio para la expresión de opiniones públicas sobre temas de interés social. Esta transformación ha impulsado el interés por realizar investigaciones sobre temas de salud mediante el análisis de comentarios en redes sociales y entornos virtuales^(6,7,8)^. En este contexto, las plataformas digitales funcionan no solo como canales de comunicación, sino también como espacios de producción simbólica donde se configuran narrativas sociales en torno a diferentes actores y profesiones.

Aunque existen estudios que analizan la imagen de la enfermería en los medios de comunicación^(9,10)^, son escasas las investigaciones que abordan este fenómeno desde la perspectiva de los contenidos generados por los usuarios en las plataformas digitales. Esta laguna limita la comprensión de cómo se configuran las representaciones públicas de la enfermería en el espacio digital, así como de los discursos que circulan en torno a la profesión.

Belting define el concepto de imagen como una simbolización personal o colectiva que se ha construido en la mente humana a partir del imaginario social del momento, proyectado por una persona o un grupo^(11)^. Esta imagen profesional, en el caso de las enfermeras, ha ido evolucionando de forma dinámica, influenciada por varios factores: desde el contacto diario con los pacientes y otros profesionales, hasta la creación de un corpus de conocimientos propios basado en el avance de la práctica basada en la evidencia^(12)^.

Esta evolución no sigue una trayectoria lineal ni es homogénea, sino que responde a contextos históricos, culturales y políticos que influyen en la forma en que la sociedad percibe la profesión.

En el ámbito de la comunicación en las redes sociales, la interacción de los usuarios con este contenido (*engagement)* puede reflejar niveles de implicación emocional, crítica o evaluativa, que ayudan a identificar elementos simbólicos y afectivos asociados a la imagen profesional de la enfermería^(13)^. Por lo tanto, en línea con los enfoques que examinan las representaciones públicas y los significados compartidos construidos en la comunicación digital cotidiana, los comentarios que los usuarios dejan en los videos relacionados con la enfermería permiten explorar percepciones, prejuicios, valoraciones y representaciones que no siempre emergen en las encuestas u otros métodos cualitativos tradicionales. Este tipo de contenido espontáneo facilita el acceso a la dimensión cotidiana del pensamiento social, donde se configuran significados compartidos, a menudo implícitos, que afectan a la forma en que se entiende y se valora el trabajo de las enfermeras.

En este sentido, este estudio contribuye a identificar estereotipos, evaluar el impacto social de las narrativas de los medios de comunicación y visibilizar la imagen profesional de las enfermeras en el entorno digital contemporáneo.

Este tipo de análisis es crucial para comprender cómo se configura la imagen social de la profesión en el espacio digital y qué implicaciones podría tener en términos de nuevas relaciones con la sociedad. Además, permite identificar tensiones entre la imagen promovida institucionalmente y las percepciones públicas expresadas libremente en las redes sociales, lo que abre oportunidades para repensar las estrategias de comunicación y educación desde el ámbito de la enfermería.

En consecuencia, y considerando el concepto de imagen propuesto por Hans Belting^(11)^ (una simbolización construida socialmente), este estudio busca analizar las representaciones públicas de la imagen de la enfermería expresadas en los comentarios de los videos de YouTube que transmiten noticias, reportajes o artículos relacionados con la profesión, publicados por cuatro canales de televisión abierta en Chile.

Este análisis se complementa con la Teoría del Cuidado Humano de Jean Watson^(14)^, que propone que el cuidado no es solo un acto técnico, sino un compromiso moral y humano con el otro, que incluye respeto, sensibilidad y presencia auténtica. Desde esta perspectiva, el cuidado también implica salvaguardar la dignidad de la profesión, dado que la relación entre la enfermería y la sociedad forma parte del entorno de cuidado. Así, comprender cómo se configura la imagen de la profesión en los espacios digitales nos permite reflexionar críticamente sobre las condiciones éticas, culturales y comunicativas que favorecen o distorsionan el reconocimiento del cuidado como valor esencial de la práctica de enfermería^(14)^.

## MÉTODO

### Tipo o Diseño del Estudio

Estudio cualitativo basado en el análisis inductivo de contenido, con el apoyo interpretativo de la Teoría del Cuidado Humano de Jean Watson^(14)^.

### Recolección de Datos

La recolección de datos se llevó a cabo a través de comentarios públicos realizados en reportajes, noticias y videos relacionados con la enfermería, publicados en las cuentas de YouTube de cuatro canales de televisión chilenos: Televisión Nacional de Chile (TVN), Canal 13 (C13), Chilevisión (CHV) y Mega, los cuales fueron seleccionados intencionadamente por ser los principales canales de televisión abiertos, con alcance nacional y los de mayor audiencia en el país^(15)^.

Se eligió YouTube como plataforma de análisis porque todos los canales tienen cuentas activas en esta red, permite subir videos más largos en comparación con otras redes sociales como Instagram y permite leer los hilos de comentarios que permanecen visibles y accesibles al público. Además, el perfil demográfico de los usuarios de los contenidos de esta plataforma social es variado, ya que es utilizada y preferida por personas de diferentes generaciones^(13)^.

### Criterios de Selección y Definición de la Muestra

Los videos se seleccionaron mediante una búsqueda en YouTube utilizando el término “enfermería” en español, combinado con el nombre abreviado de cada uno de los cuatro canales de televisión, junto con la palabra “Chile” en el caso de dos emisoras, para evitar confusiones con canales del mismo nombre de otros países.

Se aceptaron variantes del término “enfermería” en los títulos de los videos, teniendo en cuenta el género y el número gramatical, excluyendo aquellos que se centraban en otras profesiones, como los técnicos o auxiliares de enfermería.

Se consideraron para la selección los primeros 20 resultados que la plataforma mostraba por defecto, basándose en su relevancia temática y en la mayor probabilidad de ser vistos por los usuarios.

De estos videos, se identificaron los cuatro con mayor número de comentarios, bajo el supuesto de que generaban el mayor interés de la audiencia. Solo uno de los canales (C13) tenía un solo video incluido, ya que los demás no presentaban comentarios. No se aplicaron criterios de exclusión relacionados con el contenido de los videos, con el objetivo de promover la diversidad temática de los comentarios analizados.

Los comentarios se extrajeron utilizando el software basado en web Export Comments^®^ (https://exportcomments.com/), exportándolos al formato Microsoft Excel^®^. Posteriormente, se anonimizaron y se almacenaron en la plataforma Mendeley Data^®^, donde están disponibles para su visualización en su idioma original^(16)^.

### Análisis

Para el análisis de los datos, el estudio utilizó un enfoque de análisis temático inductivo, basado en la metodología propuesta por Elo y Kyngäs^(17)^. Siguiendo las etapas definidas por los autores, la fase de preparación comenzó con la selección de las unidades de análisis, correspondientes a los comentarios publicados en noticias e informes de YouTube; durante esta fase, dos investigadores leyeron repetidamente los comentarios, asignando significado, relevancia y valor a cada unidad de análisis.

En la fase de organización, se utilizó un enfoque de análisis de contenido inductivo, que incluyó la codificación abierta, la creación de categorías y la abstracción de datos. En la codificación abierta, ambos investigadores leyeron conjuntamente los comentarios, tomando notas y generando categorías preliminares basadas en los temas emergentes. Posteriormente, se eliminaron las categorías repetitivas y las restantes se agruparon en categorías similares o de nivel superior, dando lugar a subcategorías. Estas subcategorías permitieron describir el fenómeno, ampliar su comprensión y contribuir a la generación de conocimiento. Para la abstracción, los investigadores asignaron un nombre y una descripción general a las principales categorías consolidadas.

Según Elo y Kyngäs^(17)^, esta fase debe prolongarse tanto como sea razonable y factible; por lo tanto, los investigadores llegaron a un consenso para determinar el punto final del proceso de abstracción.

Todo el proceso de codificación, organización y análisis de los datos se llevó a cabo utilizando ATLAS.ti^®^ versión 25, lo que permitió sistematizar la información textual, organizar las categorías emergentes y facilitar la trazabilidad cualitativa.

Es importante señalar que, al final del proceso de análisis, los investigadores mejoraron la redacción, la gramática y adaptaron las expresiones idiomáticas locales presentes en los comentarios sin alterar su significado, para facilitar su comprensión. Aunque el análisis temático se llevó a cabo utilizando un enfoque inductivo, la interpretación de los resultados se enriqueció aún más con elementos de la Teoría del Cuidado Humano de Jean Watson^(14)^.

Este marco permitió una comprensión más profunda de las dimensiones éticas, relacionales y humanas de las representaciones identificadas, lo que permitió una lectura crítica de cómo los discursos sociales expresados en los comentarios refuerzan o erosionan los valores fundamentales del cuidado, como la empatía, la dignidad, la sensibilidad y la presencia auténtica, que se consideran fundamentales para la práctica de la enfermería.

Esta investigación cumplió con los Criterios Consolidados para la Presentación de Informes de Investigación Cualitativa (Consolidated Criteria for Reporting Qualitative Research - COREQ)^(18)^.

### Aspectos Éticos

Debido a la metodología de investigación empleada y al carácter público tanto de YouTube como de los comentarios analizados, el estudio no requirió la aprobación de un Comité de Ética Científica. Sin embargo, para proteger la identidad de los comentaristas, los investigadores anonimizaron los perfiles y no se pusieron en contacto con ellos.

## RESULTADOS

En esta sección se presentan los resultados del análisis temático de los comentarios realizados en vídeos relacionados con la enfermería de los cuatro principales canales de televisión de Chile. Se incluyeron un total de 13 vídeos, de los que se extrajeron 1018 comentarios que se sometieron a análisis. Las características de los vídeos incluidos se describen en la Tabla 1.

**Tabla 1:** Videos de YouTube incluidos por canales abiertos de TV – Chile, 2025.

Estrategia de búsqueda	Título del video en lenguaje original	Links	Comentarios	Fecha de exportación
“Enfermería TVN Chile”	Enfermero formalizado por brutal golpiza a conserje	https://www.youtube.com/watch?v=80Mz3nIS7h0	n = 30	06/12/2025
	Federación de enfermeros pide cambiar el manejo de la pandemia: "estamos sobreexplotados"	https://www.youtube.com/watch?v=4tCq_Bi6d3A	n = 33	06/12/2025
	Denuncian que enfermera utilizó la misma jeringa para vacunar a varias personas	https://www.youtube.com/watch?v=rpyXHT3yfEs&t=2s	n = 33	06/12/2025
	Grave agresión: Paciente habría tosido en la cara de enfermera	https://www.youtube.com/watch?v=MdeQY1hXDbQ	n = 56	06/12/2025
“Enfermería CHV”	FISCAL ENTREGA DETALLES tras detención de sospechosos de ataque a enfermera	https://www.youtube.com/watch?v=BxihWaDwOcY	n = 93	06/12/2025
	Habla Pola Álvarez, la enfermera apuñalada en Las Condes: “Recuerdo todo”	https://www.youtube.com/watch?v=OQuH1in1NJA	n = 116	06/12/2025
	REVELAN CONVERSACIÓN clave entre imputados previo al ataque a enfermera: “La Pola es esa, la rucia”	https://www.youtube.com/watch?v=3QHygTGQsu0	n = 142	06/12/2025
	IMPACTANTE VUELCO: Ex funcionaria y su pareja detenidos por ataque a enfermera	https://www.youtube.com/watch?v=837A5WXJ_Y0	n = 246	06/12/2025
“Enfermería Mega”	¡Heroicas! Enfermeras salvan a recién nacidos en terremoto	https://www.youtube.com/shorts/Szd2mAMPZmw	n = 8	06/12/2025
	Personalidad narcisista: El perfil de la enfermera imputada por crimen de Pola Álvarez	https://www.youtube.com/watch?v=2slYQsqNIH8	n = 9	06/12/2025
	Dos atacantes de enfermera son declarados culpables	https://www.youtube.com/watch?v=PMi1gb2t81w	n = 16	06/12/2025
	Conmovedor mensaje de enfermera emociona a ministros y Presidente en La Moneda	https://www.youtube.com/watch?v=8OrbfU001YY	n = 205	06/12/2025
“Enfermería Canal 13 Chile”	Enfermera chilena en Italia y la lucha contra el coronavirus	https://www.youtube.com/watch?v=vTo5E_NUf88	n = 31	06/12/2025

El análisis temático permitió identificar dos categorías principales: representaciones públicas que violan la ética del cuidado humano en la enfermería y representaciones públicas que refuerzan la ética del cuidado humano en la enfermería. Las categorías que se presentan a continuación reflejan los patrones de significado que surgen del discurso público expresado en los comentarios de YouTube. Estas categorías no representan representaciones sociales teóricas, sino representaciones públicas y construcciones evaluativas identificadas mediante un análisis inductivo del contenido. Las categorías y subcategorías emergentes se representan en la Figura 1.

**Figura 1:** Conceptualización de las categorías y subcategorías que surgen del análisis de contenido.

### Representaciones Públicas Que Vulneran la Ética del Cuidado Humano en la Enfermería

Desde la perspectiva del cuidado transpersonal y la ética del cuidado propuesta por Jean Watson, estas representaciones muestran una desconexión entre los principios fundamentales del cuidado profesional y las imágenes sociales que circulan en los espacios digitales. Watson concibe el cuidado como un acto moral y relacional que se expresa a través de la empatía, la compasión, el respeto por la dignidad y la presencia auténtica. Por lo tanto, las representaciones públicas que deshumanizan a los profesionales de la enfermería señalan una ruptura en la relación entre el cuidado y la cultura. De esta categoría surgieron cinco subcategorías.

#### Violación del Respeto Mutuo y la Relación de Cuidado

Watson sostiene que el cuidado auténtico se basa en el respeto y la igualdad^(14)^. En este caso, los comentarios refuerzan una imagen autoritaria que contradice el ideal ético del cuidado.

*“Quítale el título por falta de ética y agresividad” (TVN)*

*“La enfermería es una profesión de egos y abusos hacia los nuevos” (CHV)*

*“Se critican y desacreditan entre ellos, arruinando por completo nuestra profesionalidad” (CHV)*

#### Abuso de la Confianza en el Vínculo entre el Cuidador y el Receptor de Cuidados

La confianza es un componente clave en el proceso de cuidado transpersonal de Watson^(14)^. Esta subcategoría muestra cómo el discurso social infunde miedo, socavando ese vínculo fundamental.

*“Revocar su título es un peligro para la sociedad” (TVN)*

*“No sabe controlar su ira; ese hombre no puede cuidar a los pacientes” (TVN)*

*“Este incidente es muy peligroso porque involucra a personas que están “preparadas o calificadas” para trabajar en el campo de la salud” (CHV)*

#### Negación de la Empatía y la Presencia Auténtica de Cuidados

Watson defiende la presencia auténtica como la base del cuidado humano^(14)^. Esta subcategoría agrupa las representaciones que niegan esta cualidad esencial, asociando la enfermería con la frialdad, la indiferencia o la deshumanización.

*“Son despiadados porque son indiferentes con los enfermos” (CHV)*

*“Pensar que estas “personas” prestan cuidados en hospitales y clínicas” (MEGA)*

*“Las enfermeras a veces son indolentes y deshumanizantes” (CHV)*

#### Deshumanización y Quiebre de la Dignidad de los Cuidadores

Aquí, se despoja al profesional de su identidad ética y humana, lo que contradice directamente los fundamentos de la teoría de Watson, que considera la dignidad un valor inalienable del cuidado^(14)^.

*“Una mujer loca, una administradora de pacientes, Dios mío” (MEGA)*

*“¿Cómo puede un criminal loco trabajar con pacientes?” (CHV)*

*“Es un criminal y una persona enferma... No es un enfermero... Debería estar en la cárcel” (TVN)*

#### Percepción de Inconsistencia Entre el ideal Ético del Cuidado y la Práctica

Aquí, los comentarios juzgan el comportamiento profesional basándose en una expectativa ética no cumplida. Desde la perspectiva de Watson, esto puede interpretarse como una desconexión entre el cuidado esperado (basado en la ética y la humanidad) y lo que se observa o se representa socialmente^(14)^.

*“Sin ética profesional” (CHV)*

*“No hay ética profesional en absoluto” (TVN)*

*“Los profesionales con ética salvan vidas humanas en todos los niveles, buen trato, juicio, humanidad, empatía... lo que en este caso es totalmente contradictorio” (CHV)*

### Representaciones Públicas Que Refuerzan la Ética del Cuidado Humano en la Enfermería

Desde la perspectiva de Watson, estos comentarios expresan una apreciación pública del cuidado como un acto ético, relacional y profundamente humano. Los discursos reconocen la enfermería no solo como una profesión técnica, sino como un compromiso moral con los demás. Surgieron dos subcategorías.

#### Reconocimiento Efectivo y Humano del Papel de la Enfermería

Estos significados se alinean con la visión de Watson del cuidado como una expresión moral, espiritual y relacional.

El cuidado como un acto altruista y ético, basado en la compasión y el amor por los demás. Para Watson, el cuidado no es solo una acción profesional, sino una expresión espiritual y moral que honra la vida de los demás^(14)^. Reconocer a las enfermeras como figuras de dedicación, protección y valentía es coherente con los profesionales que se conectan auténticamente con los demás, sin esperar recompensa, promoviendo la curación más allá del cuerpo físico.

*“Dan su vida por las personas” (Mega)*

*“Trabajan arriesgando sus vidas para protegernos” (TVN)*

*“Un corazón gigante y valiente, decidido a darlo todo por los demás sin esperar nada a cambio” (Mega)*

#### Valoración Positiva del Compromiso y la Dedicación Profesional

Aquí, el público ancla la enfermería en valores como la sensibilidad, la presencia y la responsabilidad. Se trata de significados culturales compartidos que dan forma al imaginario colectivo sobre la profesión y que resuenan con los procesos de Caritas descritos por Watson. Compromiso ético, técnico y emocional implícito en la práctica profesional de la enfermería. La referencia a “estar en manos” de una enfermera se refiere al concepto de presencia auténtica, en el que el profesional está completamente disponible para cuidar de los demás^(14)^. Del mismo modo, la “sensibilidad” alude a los aspectos centrales del proceso Caritas.

*“¿Cómo puede la gente imaginar que una enfermera va a reutilizar jeringas?” (TVN)*

*“El personal de enfermería debe tener una gran sensibilidad hacia sus semejantes” (CHV)*

*“Siempre admiré a las enfermeras porque mi vida estaba en sus manos cada vez que me ponían una vía intravenosa” (CHV)*

## DISCUSIÓN

Si bien existen estudios que analizan la imagen social de las enfermeras en los medios tradicionales^(19,20,21,22)^, las nuevas formas de interacción digital, como los sitios web, los blogs y las plataformas de redes sociales como YouTube y X, han adquirido una influencia relevante^(23)^. En este contexto, considerando que el 66% de la población mundial, es decir, más de 5350 millones de personas, son usuarios de Internet^(24)^, este estudio ofrece un enfoque novedoso, centrándose específicamente en el análisis de los comentarios de los usuarios en una red social digital, lo que permite acceder a las percepciones espontáneas y sin mediar del público.

En cuanto a los resultados, las representaciones negativas, que fueron predominantes, ofrecen una visión estigmatizada de la imagen profesional de las enfermeras. Este hallazgo es coherente con una revisión de alcance, que indica que los medios de comunicación tienden a difundir predominantemente una imagen negativa de la profesión^(10)^. Esta percepción de la imagen profesional constituye un poderoso mecanismo que influye en aspectos clave como la autoestima y la autoridad de las enfermeras, así como en la contratación y retención de personal en el sector sanitario^(25,26,27)^ y también, en su intención de abandonar la profesión^(28)^. Incluso, el Consejo Internacional de Enfermeras (CIE) destacó la importancia de esta cuestión en su informe global, que refleja cómo la falta de reconocimiento y el deterioro de la imagen social contribuyen a la escasez de mano de obra y a la alta rotación en todo el mundo^(29)^.

Por el contrario, las representaciones positivas de la imagen profesional, aunque presentes, surgen con menos frecuencia y en particular, en vídeos en los que las enfermeras son representadas como figuras de heroísmo y sacrificio. En cuanto a esta imagen positiva, algunos autores sostienen que, en general, la sociedad atribuye mayor importancia a características como la amabilidad y la cercanía, identificando la vocación como el núcleo central de la enfermería^(30)^. En consonancia con esto, Jean Watson sugiere que la atención profesional se basa en una disposición auténtica hacia los demás, sustentada por el amor, el compromiso y la intención de sanar: “el acto de cuidar es una expresión moral y espiritual, no meramente técnica”^(14)^.

Una imagen positiva de una profesión en la sociedad puede conferir poder, reconocimiento y estatus, lo que contribuye al posicionamiento simbólico y moral de la profesión en la sociedad^(19)^. En términos de cuidado humano, esa imagen pública también refuerza la legitimidad ética de la enfermería, ya que, como señala Watson, “el cuidado auténtico se basa en el respeto mutuo y el reconocimiento de la humanidad compartida”^(14)^. Por lo tanto, presentar una imagen pública positiva de la enfermería es una cuestión urgente que debe abordarse para crear una fuerza laboral de enfermería empoderada^(31)^ y con mayor capacidad de liderazgo^(32)^.

La confianza social en la función de la enfermería, cuando se ve erosionada por representaciones estigmatizantes, no solo debilita la imagen profesional, sino que, desde una perspectiva humanista, daña el vínculo esencial entre el cuidador y el receptor^(33)^.

Watson sostiene que “la confianza es la base sobre la que se construye toda relación de cuidado” y que, sin ella, el proceso de curación se ve amenazado, lo que contribuye al posicionamiento simbólico y moral de la profesión en la sociedad^(14)^. Aunque es solo una de las muchas teorías de enfermería que reflejan el cuidado, el énfasis de Watson en las relaciones auténticas en los entornos de curación refuerza la idea de que la enfermería no es solo una profesión, sino una vocación de servir a la humanidad con empatía y amor^(34)^.

En este escenario desafiante, es importante que las enfermeras y las organizaciones profesionales que las representan generen instancias de interacción activa, con el propósito de proyectar y transmitir una imagen más realista de la profesión^(35)^. Al participar activamente en medios como los canales de televisión, las enfermeras pueden dar a conocer su función, su valor y su importante contribución a los sistemas de salud, y reducir la transmisión de contenidos que afectan negativamente a la imagen que la sociedad tiene de la profesión. Sin embargo, para Watson, esta tarea no es solo comunicativa, sino también ética, ya que implica hacer visible la atención como una práctica humana, relacional y digna, que “reconoce al otro como un ser único e irrepetible, merecedor de respeto, compasión y presencia auténtica”^(14)^.

Por último, a pesar de su rigor metodológico, este estudio tiene algunas limitaciones. Estas se relacionan principalmente con las características de los videos seleccionados, que en su mayoría corresponden a reportajes y noticias con un enfoque negativo sobre la profesión, y podrían haber influido en el contenido de los comentarios analizados. Además, aunque los resultados proporcionan una visión inicial de la imagen pública de la enfermería en esta plataforma, las investigaciones futuras deberían ampliarse a otras redes sociales, como Instagram, X o Facebook, para enriquecer aún más la comprensión de la representación social de la enfermería en el entorno digital.

## CONCLUSIÓN

Este estudio nos ha permitido analizar las representaciones públicas y los discursos que circulan sobre la enfermería en una plataforma digital de gran alcance como YouTube, basándonos en los comentarios espontáneos de los usuarios sobre noticias relacionadas con la profesión. Los resultados revelan la coexistencia de representaciones contradictorias: por un lado, una imagen positiva vinculada a la dedicación, la sensibilidad y el compromiso ético, valores profundamente ligados al cuidado humano y ampliamente valorados por la sociedad; por otro, una narrativa predominante de desconfianza, estigmatización y cuestionamiento del papel profesional, que afecta a la confianza pública y al estatus social de las enfermeras. Esta dualidad refleja tensiones persistentes en la percepción pública de la profesión y abre un campo de reflexión sobre los factores sociales, culturales y éticos que influyen en su reconocimiento. En este contexto, es esencial promover estrategias que refuercen la visibilidad de la atención como una práctica ética y humana, restaurando la dignidad y la confianza en la enfermería, tal como propone la Teoría del Cuidado Humano de Jean Watson, contribuyendo así al empoderamiento y la apreciación social de la profesión.

## Data Availability

El conjunto de datos que respaldan las conclusiones de este estudio está disponible previa solicitud al autor correspondiente.
